# Endoplasmic Reticulum Aminopeptidase 1 Is Involved in Anti-viral Immune Response of Hepatitis B Virus by Trimming Hepatitis B Core Antigen to Generate 9-Mers Peptides

**DOI:** 10.3389/fmicb.2022.829241

**Published:** 2022-05-04

**Authors:** Huanhuan Liu, Bingqi Hu, Junfeng Huang, Qin Wang, Feier Wang, Faming Pan, Liwen Chen

**Affiliations:** ^1^Department of Laboratory Medicine, Second Hospital of Anhui Medical University, Hefei, China; ^2^Department of Epidemiology and Biostatistics, School of Public Health, Anhui Medical University, Hefei, China

**Keywords:** hepatitis B, endoplasmic reticulum aminopeptidase 1, major histocompatibility complex class I, antigen presentation, immune response

## Abstract

Endoplasmic reticulum aminopeptidase 1 (ERAP1) is a processing enzyme of antigenic peptides presented to major histocompatibility complex (MHC) class I molecules. ERAP1-dependent trimming of epitope repertoire determines an efficacy of adoptive CD8^+^ T-cell responses in several viral diseases; however, its role in hepatitis B virus (HBV) infection remains unknown. Here, we show that the serum level of ERAP1 in patients with chronic hepatitis B (CHB) (*n* = 128) was significantly higher than that of healthy controls (*n* = 44) (8.78 ± 1.82 vs. 3.52 ± 1.61, *p* < 0.001). Furthermore, peripheral ERAP1 level is moderately correlated with HBV DNA level in patients with CHB (*r* = 0.731, *p* < 0.001). HBV-transfected HepG2.2.15 cells had substantially increased ERAP1 expression and secretion than the germline HepG2 cells (*p* < 0.001). The co-culture of ERAP1-specific inhibitor ERAP1-IN-1 pretreated HepG2.2.15 cells or *ERAP1* knockdown HepG2.2.15 cells with CD8^+^ T cells led to 14–24% inhibition of the proliferation of CD8^+^ T cells. Finally, liquid chromatography tandem mass spectrometry (LC-MS/MS) test demonstrated that ERAP1-IN-1 blocks completely the production of a 9-mers peptide (30–38, LLDTASALY) derived from Hepatitis B core antigen (HBcAg). The predictive analysis by NetMHCpan-4.1 server showed that human leukocyte antigen (HLA)-C*04:01 is a strong binder for the 9-mers peptide in HepG2.2.15 cells. Taken together, our results demonstrated that ERAP1 trims HBcAg to produce 9-mers LLDTASALY peptides for binding onto HLA-C*04:01 in HepG2.2.15 cells, facilitating the potential activation of CD8^+^ T cells.

## Introduction

World Health Organization (WHO) Global hepatitis report, 2017 has shown that in 2015, 257 million people were estimated who have been chronically infected with hepatitis B virus (HBV) and more than 887,000 patients die annually due to chronic HBV infection (CHB)-related diseases worldwide (World Health Organization, 2018; Stinco et al., 2021). The risk of CHB after acute infection is reduced to < 5% for immunocompetent adults (Chen and Tian, 2019). A vigorous CD8^+^ T-cell response that exhibits antiviral activity by producing interferon-γ (IFN-γ) and tumor necrosis factor-α (TNF-α) or by directly killing the infected hepatocytes is generated to control and clear HBV (Guidotti et al., 1999; Maini et al., 2000; Guidotti and Chisari, 2001; Thimme et al., 2003).

A fundamental component of host immunity against viral infection relies on the presentation of endogenously derived peptides by major histocompatibility complex class I (MHC-I) molecules (Watts and Powis, 1999). These peptide MHC-I (pMHC-I) complexes are presented on the cell surface and recognized by CD8^+^ cytotoxic T lymphocytes (CTLs) (Chang et al., 2005; Cresswell, 2019; Weimershaus et al., 2019). Antigenic peptides are generated by the degradation of proteasome, translocation into the endoplasmic reticulum (ER) lumen by the transporter associated with antigen processing (TAP), and trimming of excess N-terminal amino acids by aminopeptidases (Alvarez-Navarro and Lopez de Castro, 2014). The endoplasmic reticulum aminopeptidase 1 (ERAP1) belongs to the oxytocinase subfamily of M1 zinc metallopeptidases and trims the N-terminus of peptides to the optimal size of 8–9 amino acids for loading onto MHC-I (Hammer et al., 2007). pMHC-I complexes are then shuttled to the cell surface for antigen presentation, which triggers antigen-specific cytolytic and effector T-cell responses. The regulation of ERAP1 expression has been shown to substantially affect the presentation of immunodominant epitopes, thereby altering the MHC I immunopeptidome profiling (York et al., 2006; Hammer et al., 2007; Cifaldi et al., 2011). However, whether ERAP1 is involved in HBV-specific immune response, namely, the antigenic peptide presentation remains unknown. In this study, we analyzed ERAP1 expression in HBV-transfected HepG2.2.15 cells and peripheral ERAP1 level in patients with CHB. We further explored the enzymatic activity of ERAP1 in HepG2.2.15 cells by the specific ERAP1 inhibitor ERAP1-IN-1, especially its effects on HBV-derived peptides and CD8^+^ T-cell stimulation. Finally, the binding of ERAP1-trimmed HBV-derived peptides with MHC class I molecules was predicted.

## Materials and Methods

### Patients and Serum Samples

Serum samples were obtained from patients with CHB (*n* = 128) and healthy controls (HC) (*n* = 44) in the Second Hospital of Anhui Medical University from June 2018 to July 2021. The baseline and laboratory data of patients with CHB and health controls are shown in Table 1. CHB was defined as the persistent presence of serum hepatitis B surface antigen (HBsAg) for > 6 months, and serum tests were performed before receiving any anti-HBV treatment. The exclusion criteria were as follows: (1) coinfection with other hepatotropic viruses (hepatitis A/C/D/E virus) or human immunodeficiency virus (HIV); (2) the coexistence of autoimmune hepatitis, alcoholic liver disease, non-alcoholic fatty liver disease, drug hepatitis, primary biliary cirrhosis, or hepatocellular carcinoma. The study was conducted in the principles of the Declaration of Helsinki and was approved by the Ethics Committee for the Second Hospital of Anhui Medical University, and all patients have signed informed consent.

**TABLE 1:** Baseline and laboratory data of patients with CHB and health controls (HC).

Characteristic	CHB (*n* = 128)	HC (*n* = 44)	*p*-value
Age (year)	38 ± 5	39 ± 7	0.136
Gender (Male/Female)	86/42	30/14	0.986
ALT (U/L)	75 (48, 119)	31 (19, 73)	<0.001
AST (U/L)	50.30 (32.00, 75.00)	21.90 (19.35, 24.80)	<0.001
TBIL (μmol/L)	25.70 (15.40, 52.10)	13.00 (7.94, 18.00)	<0.001
ALB (g/L)	41.13 ± 6.23	47.52 ± 3.06	0.035
GGT (IU/L)	57.92 ± 7.64	23.56 ± 4.17	<0.001
HBV genotype		–	–
A	0		
B	31	–	–
C	97	–	–
D	0		
HBV DNA (Log_10_ IU/ml)	2.31 (2.27, 3.70)	–	–
HBsAg (IU/ml)	2,576 (739, 6,881)	–	–
HBeAg (S/CO)	743.52 (236.19, 3286.50)	–	–

### Routine Laboratory Tests

Fasting serum level of alanine aminotransferase (ALT, IU/L), aspartate aminotransferase (AST, IU/L), total bilirubin (TBIL, μmol/L), albumin (ALB, g/L), and gamma-glutamyl transpeptidase (GGT, IU/L) was detected by AU5800 biochemistry analyzer (Beckman Coulter, CA, United States). HBV genotyping was based on line probe assays and genotype-specific PCR (Fosun Long March Medical Science, Shanghai, China) according to the manufacturer’s instructions. The supernatants from HepG2 and HepG2.2.15 cells treated or left untreated with ERAP1-IN-1 (50 μm) (HY-133125, MedChem Express, New Jersey, United States) were collected 72 h later. HBsAg and HBeAg in the sera and supernatants were measured using commercially available kits with chemiluminescence apparatus (ARCHITECT i2000SR; Abbott diagnostics, IL, United States). HBV viral loads in the sera and supernatants were measured using the real-time polymerase chain reaction (Mx3000p, Agilent Technologies, CA, United States) according to the manufacturer’s instructions.

### *In vitro* Enzymatic Assay

The inhibitory potency of ERAP1-IN-1 for ERAP1 was determined using an established fluorogenic assay as previously described (Papakyriakou et al., 2013).

### Cell Culture

HepG2. 2.15 cells that are characterized by having stable HBV expression and replication and the germline HepG2 cells were purchased from Shanghai Fuheng Biotechnology. Both cell lines were cultured in Dulbecco’s modified eagle medium (DMEM) containing stable glutamine, supplemented with 10% heat-inactivated fatal bovine serum (FBS) (Gibco) and penicillin (100 IU/ml)/streptomycin (100 μg/ml), and incubated at 37^°^C, 5% CO_2_. Both cell lines were authenticated using short tandem repeat (STR) analysis in combination with sex-typing gene amelogenin detection and compared with DSMZ STR cell line profiles before the use.

### Western Blotting

HepG2 and HepG2.2.15 cells were lysed in a radio immunoprecipitation assay (RIPA) buffer supplemented with proteinase inhibitors. About 40 μg of proteins was loaded and separated on sodium dodecyl sulfate–polyacrylamide gel electrophoresis (SDS-PAGE). The protein was then transferred onto a polyvinylidene fluoride (PVDF) membrane, blocked in 5% (w/v) non-fat milk, and incubated with the primary antibodies. The source of the primary antibody was anti-ARTS1/ERAP1 antibody (Abcam, ab124669) and secondary antibody was the Goat Anti-rabbit antibody (Abcam, ab6721). Finally, the protein blots were visualized using enhanced chemiluminescent (ECL) system (Thermo Fisher Scientific, United States). The expression levels of ERAP1 were assessed using ImageJ software (v1.8.0, National Institutes of Health) and were normalized against β-actin.

### Enzyme-Linked Immunosorbent Assay

The peripheral ERAP1 was detected using enzyme-linked immunosorbent assay (ELISA) kits (OM534481, Omni mAbs, United States) according to the manufacturer’s instructions.

### Gene Silencing of Endoplasmic Reticulum Aminopeptidase 1

The small interfering RNA target ERAP1 (Si-ERAP1) and the negative control siRNA (SiControl) oligos were synthesized by Jima Biotechnology (Shanghai, China). The sequences of the siRNA-ERAP1 were as follows: siERAP1 #1, 5′-GGG CGA GUC UCA UUA ACA ATT-3′ and siERAP1 #2, 5′-UUG UUA AUG AGA CUC GCC CTT-3′. The siControl oligos were as follows: siControl #1, 5′-UUC UCC GAA CGU GUC ACG UTT-3′ and siControl #2, 5′-ACG UGA CAC GUU CGG AGA ATT-3′. Lipofectamine 3,000 reagent (L3000015, Invitrogen, United States) was used to perform transfection according to the manufacturer’s protocol. The siRNA-transfected and mock HepG2.2.15 cells were incubated at 37^°^C in 5% CO_2_, and ERAP1 knockdown was verified at 48 h post-transfection *via* western blotting analysis.

### CD8^+^ T-Cell Isolation

Peripheral blood mononuclear cells (PBMCs) were prepared from the heparinized venous blood of healthy volunteers by Ficoll-paque plus (GE, United States) density gradient centrifugation. Human naïve CD8^+^ T cells were sorted by positive magnetic-activated cell sorting (MACS, Miltenyi Biotec, United States) according to the manufacturer’s instructions. The cell purity was confirmed by flow cytometry using an anti-mouse CD8 antibody (eBioscience, CA, United States).

### Co-culture of CD8^+^ T Lymphocytes With HepG.2.2.15 Cells

HepG2.2.15 cells were treated or left untreated by ERAP1-IN-1 (50 μm) for 72 h followed by the treatment with mitomycin C (10 μg/ml) for 2.5 h. Cells were washed 5 times with PBS to remove residual ERAP1-IN-1 and mitomycin C and resuspended in DMEM at a final concentration of 1 × 10^4^/ml. The isolated CD8^+^ T lymphocytes and HepG2.2.15 cells (10:1) were seeded in the 96-well plate, supplemented with recombinant human IL-2 (200 U/ml), and cultured for 14 days. The proliferation of CD8^+^ T cells was determined by WST-8/CCK-8 at 0, 3, 5, 7, 10, and 14 days. Briefly, 10 μl of the WST-8/CCK-8 solution (Biosharp, Shanghai, China) was added to each well and incubated for 4 h. Finally, the absorbance was measured at 450 nm.

### ERAP1-IN-1 Cytotoxicity Assay

To evaluate ERAP1-IN-1 cytotoxicity against HepG2.2.15 cells, 100 μl (10^4^/ml) cell suspension was dispensed into 96-well plate, and varying concentrations (0, 20, 40, 60, 80, and 100 μm) of ERAP1-IN-1 was added and cultured for 72 h. About 10 μl of the WST-8/CCK-8 solution (Biosharp, Shanghai, China) was added to each well and incubated for 4 h, and the absorbance was measured at 450 nm. All experiments were performed at least three times and the relative cell viability (%) was expressed as a percentage relative to untreated control cells. Cell viability (%) = OD value of ERAP1-IN-1 group/OD value of control group × 100%.

### Protein Extraction and Digestion

After 72 h of treatment with ERAP1-IN-1, HepG2.2.15 cells were harvested for peptidome analysis. SDS with DTT (SDT) buffer (4% SDS, 100 mM Tris-HCL, 1 mM DTT, pH7.6) was used for cell lysis and protein extraction. The extracted protein was quantified with the BCA (bicinchoninic acid) Protein Assay Kit (Bio-Rad, United States). The protein suspensions were digested with 4 μg trypsin (Promega, Madison, United States) in 40 μl 25 mM NH_4_HCO_3_ buffer overnight at 37°C. The digested peptides of each sample were desalted on C18 Cartridges (Empore™ SPE Cartridges C18, Sigma), concentrated by vacuum centrifugation, and reconstituted in 40 μl of 0.1% (v/v) formic acid.

### Mass Spectrometry Analysis

Liquid chromatography tandem mass spectrometry analysis was performed on a timsTOF Pro mass spectrometer (Bruker) that was coupled to Nanoelute (Bruker Daltonics). Peptides were separated using a linear gradient of buffer (84% acetonitrile and 0.1% formic acid) for 120 min at a flow rate of 300 nl/min and a Thermo Scientific reverse-phase nano Easy-spray column (Thermo Scientific PepMap C18, 2 μm particle size, 100 A pore size, 75 μm i.d). The mass spectrometer was operated in positive ion mode. The mass spectrometer collected ion mobility MS spectra over a mass range of m/z 100--1,700 and 1/k0 of 0.6--1.6 and then performed 10 cycles of PASEF MS/MS with a target intensity of 1.5 k and a threshold of 2,500. The dynamic exclusion was set to 24 s. The MS raw data for each sample were combined and searched using the MaxQuant (1.5.3.17) software for the identification and quantitation analysis. The integration of each peptide and protein signal on the LC-MS was calculated by the chromatography based on MS1. Precursor ion mass and fragment mass tolerance was set to 20 ppm, FDR was set to 0.01, peptide-spectrum matching FDR was set to 0.01, and oxidation (Met) was used as the variable modifications. Any identification from the reverse database and known contaminants was eliminated. The mass spectrometry proteomics data have been deposited to the ProteomeXchange Consortium^1^ *via* the iProX partner repository with the dataset identifier PXD031858.

### Affinity of Peptides to Major Histocompatibility Complex Class I Molecules Predicted by NetMHCpan

We adopted NetMHCpan-4.1 server^2^ to predict the binding of peptides to any MHC molecule of known sequence using artificial neural networks (ANNs). The protein sequence of HBcAg (GenBank: AXM44938.1, 185aa) was inputted in FASTA format and peptide length of 9-mers was selected for analysis. Being an automated method, NetMHCpan-4.1 is trained on a combination of more than 850,000 quantitative binding affinities (BA) and mass spectrometry eluted ligands (EL) peptides. The prediction outputs used for each MHC allele include score_EL and % Rank_EL. The former is raw prediction score, whereas % Rank_EL denotes the rank of the predicted binding score compared to a set of random natural peptides. This measure is not affected by an inherent bias of certain molecules toward higher or lower predicted affinities. The bind level includes strong binder (SB) and weak binder (WB). Strong binders are defined as having % Rank_EL < 0.5 and weak binders with % Rank_EL < 2.

### Statistical Analysis

All data were processed and analyzed by SPSS 24.0 and GraphPad Prism 7.0. Normal data were described with mean and standard deviation (SD), and comparisons between two groups were performed using two-tailed Student’s *t*-tests. Data of abnormal distribution were expressed as a median with a range of lower–upper quartiles (between 25th and 75th percentiles) and analyzed by Mann–Whitney U test. Correlations between variables were evaluated using the Spearman’s rank correlation test. *p*-value < 0.05 was considered as significantly different.

## Results

### Hepatitis B Virus Infection Upregulates Endoplasmic Reticulum Aminopeptidase 1 Expression

**FIGURE 1:**
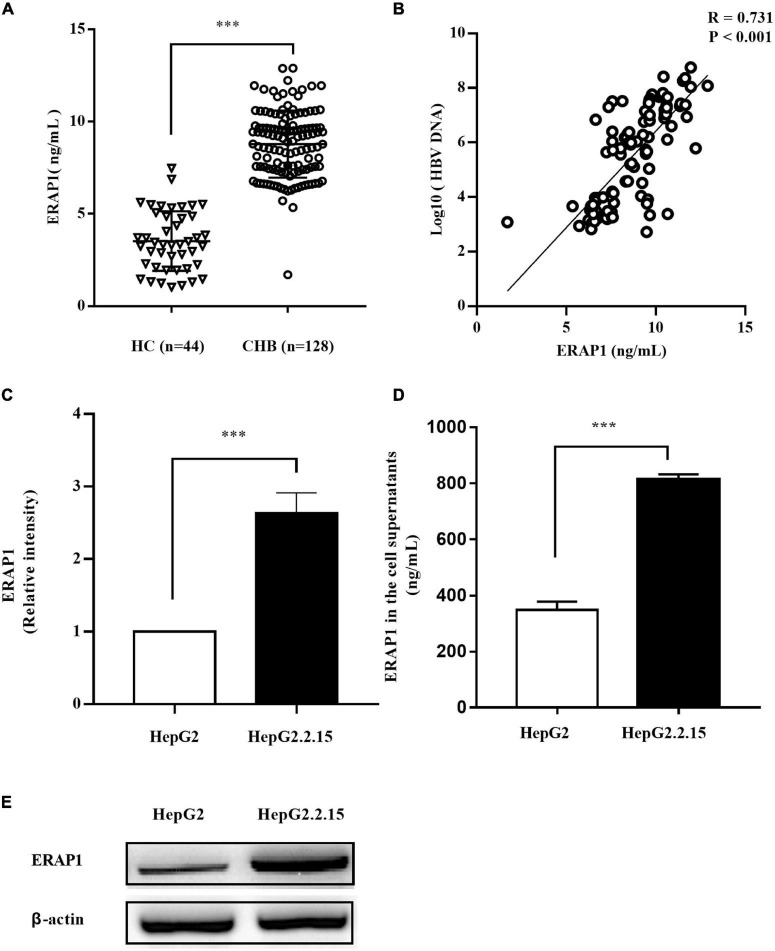
HBV infection promotes ERAP1 expression and secretion. **(A)** the peripheral level of ERAP1 in patients with CHB (*n* = 128) and HC (*n* = 44) was detected by ELISA. **(B)** Pearson’s correlation analysis between sera ERAP1 and HBV DNA of patients with CHB. **(C,E)** ERAP1 expression and quantitative analysis in HepG2.2.15 cells and the germline HepG2 cells. **(D)** The amounts of ERAP1 in supernatants ofHepG2.2.15 cells and the germline HepG2 cells. Data are presented as the mean ± SD of three independent experiments. CHB, chronic hepatitis B; HC, healthy control. ***p < 0.001.

Endoplasmic reticulum aminopeptidase 1 is usually retained in the lumen of ER and was therefore named for the localization. However, it could be secreted into the extracellular milieu in response to inflammatory stimuli, although the precise molecular mechanisms behind the secretion remain unclear (Tsujimoto et al., 2020). We therefore analyzed peripheral ERAP1 level in patients with CHB. As shown in Figure 1A, the serum level of ERAP1 in patients with CHB (*n* = 128) was significantly higher than that of healthy controls (*n* = 44) (8.78 ± 1.82 ng/ml vs. 3.52 ± 1.61 ng/ml, *p* < 0.001). Furthermore, peripheral ERAP1 level was moderately correlated with HBV DNA levels in patients with CHB (Figure 1B, *r* = 0.731, *p* < 0.001). HBV-transfected HepG2.2.15 cells had substantially increased ERAP1 expression than the germline HepG2 cells (*p* < 0.0001, two-tailed unpaired Student’s *t*-tests) (Figures 1C,E). Meanwhile, ERAP1 level in the cell supernatants was detected by ELISA. We found that the ERAP1 secretion was also considered excessive when the intracellular ERAP1 protein levels were elevated (Figure 1D). These results indicate that HBV infection upregulates ERAP1 expression from cellular to organismic level.

### Endoplasmic Reticulum Aminopeptidase 1 Promotes Hepatitis B Virus-Specific CD8^+^ T-Cell Proliferation

Endoplasmic reticulum aminopeptidase 1 acts as a final processing enzyme of antigenic peptides presented to MHC class I molecules (Wearsch and Cresswell, 2008). We therefore investigated the effects of HBV-induced ERAP-1 upregulation on CD8^+^ T lymphocytes. We used ERAP1-IN-1, a recently discovered non-peptide compound, which competitively inhibits ERAP1 activity toward a nonamer peptide suitable for MHC class I presentation (Maben et al., 2020). The inhibitory effect of ERAP1-IN-1 is dose-dependent, and we choose the working concentration at 50 μm that exhibited specific inhibition of peptide presentation in the cellular context (Maben et al., 2020). The cell viability of HepG2.2.15 cells was not affected by ERAP1-IN-1 at 50 μm for 72 h, even though a significant decrease was observed both at 80 and 100 μm (Supplementary Figure 1). In this study, we also demonstrated dose-dependent inhibition of ERAP1-IN-1 on the *in vitro* enzymatic activity of recombinant ERAP1 (Supplementary Figure 2). Additionally, being a newly reported inhibitor, the effects of ERAP1-IN-1 on HBV replication need to be assessed. Our results showed that HBsAg, HBeAg, and HBV-DNA in the supernatants of HepG2.2.15 treated with ERAP1-IN-1 (50 μm for 72 h) have no significant difference with that of HepG2.2.15 cells left untreated (Supplementary Figure 3). Besides, *ERAP1* knockdown HepG2.2.15 cells were used paralleled to ERAPI-IN-1 to verify the effects of the inhibitor.

**FIGURE 2:**
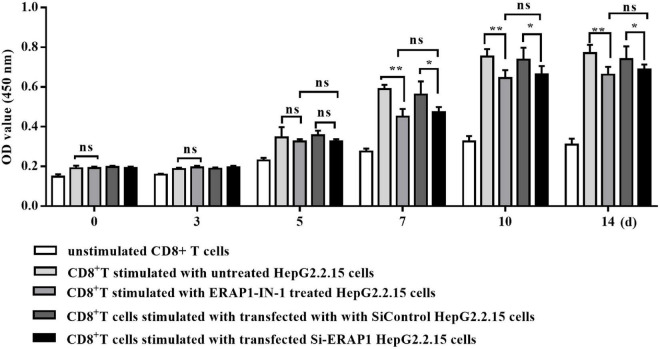
ERAP1-IN-1 or EARP1 knockdown restrains the stimulating ability of HepG2.2.15 cells to CD8^+^ T cells. HepG2.2.15 cells were pretreated with ERAP1-IN-1 (50 μm) for 72 h, or siERAP1 HepG2.2.15 cells were treated with mitomycin C (10 μg/ml) for further 2.5 h and then co-cultured with isolated CD8^+^ T cells (1:10). The proliferation of CD8^+^ T cells was detected by CCK-8 (OD 450 nm) 0, 3, 5, 7, 10, and 14 days later after co-culturing. ERAP1-IN-1 untreated or mock HepG2.2.15 cells were used as stimulator control, and unstimulated CD8^+^ T cells were used as negative control. ns, no significance; *P < 0.05; **p < 0.01.

The co-culture of ERAP1-IN-1 pretreated or ERAP1 knockdown HepG2.2.15 cells with CD8^+^ T cells significantly reduced proliferation of CD8^+^ T cells after time periods of 7, 10, and 14 days in culture, as compared to untreated and mock HepG2.2.15 cells, respectively (Figure 2). The percent inhibition calculated for CD8^+^ T cells ranged from 14% to 24%. These results strongly suggested that ERAP1 promotes anti-HBV immune response in the context of the presentation of antigenic peptides by MHC class I molecules to stimulate CD8^+^ T lymphocytes.

### Endoplasmic Reticulum Aminopeptidase 1 Regulates Peptide Distribution in Hepatitis B Virus-Infected HepG2.2.15 Cells

**FIGURE 3:**
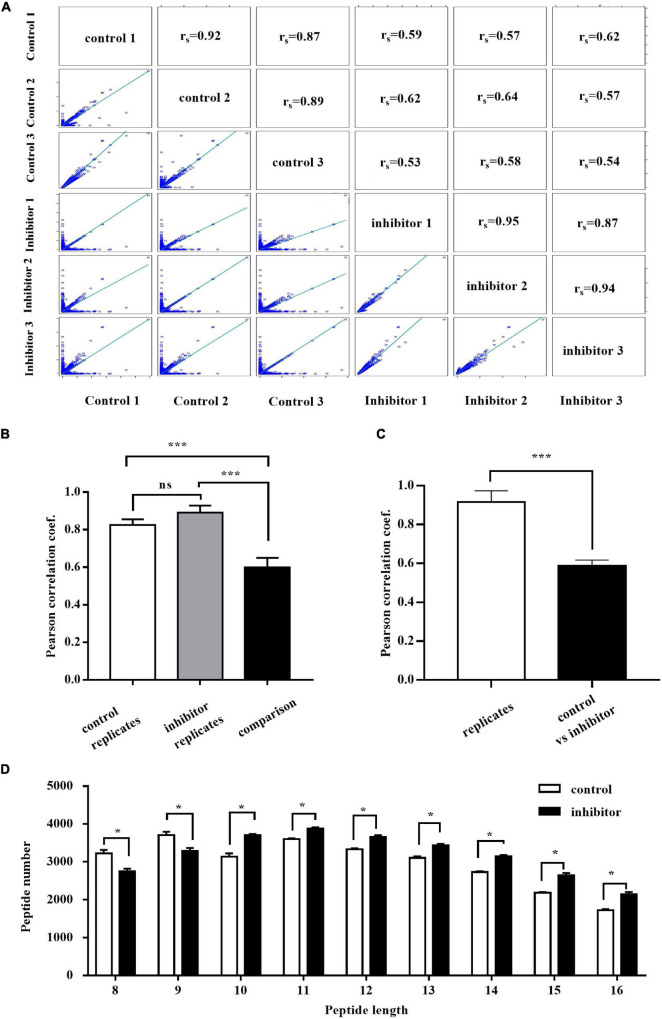
ERAP1-IN-1 treatment affects (8–16)-mers peptides distribution in HepG2.2.15 cells. **(A)** Peptide intensity correlations between different experiments and replicates. The calculated Pearson coefficient is indicated in each panel. **(B)** Average Pearson coefficient of correlations between control replicates, inhibitor replicates, and control vs. inhibitor experiments. **(C)** Average Pearson coefficient of correlations between replicates of the same condition and control vs. inhibitor. **(D)** The peptide length distribution of 8–16 residues in the control and inhibitor groups detected by LC/LC-MS. LFQ, label-free quantification. *P < 0.05; ***P < 0.001.

It has been well characterized that ERAP1 has length specificity unique among aminopeptidases, efficiently trimming longer (9–16 residues) peptides to 8–9 residues that match the length preferences of MHC-I (Chang et al., 2005). Thus, we analyzed the distribution of the peptide with size range of 8–16 by LC-MS/MS in HepG2.2.15 cells treated with ERAP1-IN-1 (50 μm) for 72 h. To have sufficient statistical power to define the inhibitor-induced changes, we performed three biological replicates for each condition. The cross-correlation analysis revealed the Pearson’s correlation coefficients of *r* > 0.85 and *r* < 0.65 between the same condition replicates and between cross-pairs (inhibitor vs. control), respectively (Figures 3A–C). Among the total peptides identified, ERAP1-IN-1-treated group had 6.24% and 4.32% reduction of the numbers of peptides with 8 and 9 residues, respectively. In contrast, all peptides > 9-mers have a number increased by a range from 3.01% to 27.14% (Figure 3D). Thus, our results demonstrated that ERAP1 regulates peptide numbers of different segments in HBV-infected HepG2.2.15 cells, leading to a significant shift of the peptides length distribution.

### Endoplasmic Reticulum Aminopeptidase 1 Trims HBcAg-Specific Peptides in Hepatitis B Virus-Infected Cells

**FIGURE 4:**
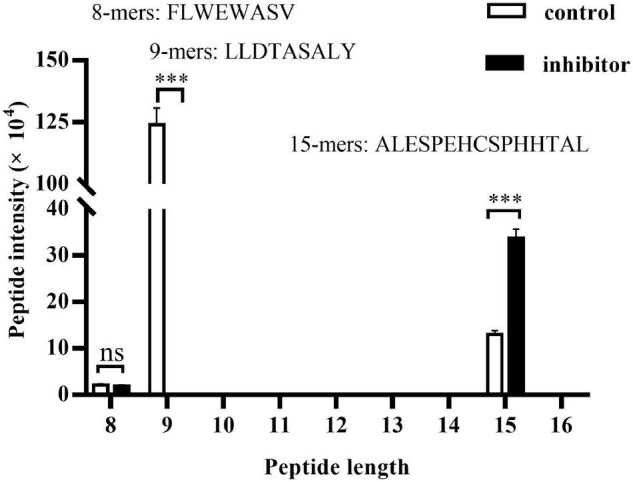
ERAP1 trims HBcAg-specific peptides in HepG2.2.15 cells. HepG2.2.15 cells were treated with ERPA1-IN-1 (50 μM) for 72 h. Cells were lysed, and the extracted protein was then analyzed by LC-MS/MS. The LFQ intensity of 8–16-mers residues was calculated and the peptides origin was identified by the MaxQuant (1.5.3.17) software *via* sequence-specific annotations based on the UniProt databases. ns, no significance; ^***^p < 0.001.

The total kinds of peptides with size range of 8–16 detected by LC/MS in HepG2.2.15 cells are more than 5,000, which derived from more than 20,000 kinds of total peptides. However, only three kinds of 8–16 residues peptides are derived from HBV genome. The peptide distribution includes one kind of 8-mers (161–168, FLWEWASV) peptide which is cleaved from HBsAg and two kinds of peptides enzymatically cut from HBcAg, 9-mers (30–38, LLDTASALY) and 15-mers (41–55, ALESPEHCSPHHTAL) (Figure 4). We further compared the intensity of the three peptides between HepG2.2.15 cells treated or left untreated with ERAP1-IN-1. The inhibitor produced a complete block of 9-mers peptide and a substantial increment of 15-mers peptide while having no remarkable effect on 8-mers. Taken together, these results suggested that ERAP1 trims HBcAg-specific peptides in HBV-infected cells.

### Prediction of the Binding of 9-mers LLDTASALY Peptide With HLA Class I Molecules

The Expert Protein Analysis System (ExPASy) databases provide a detailed description of HLA allotypes expressed by the HepG2 cells.^3^ Table 2 shows the bind level output of six HLA I alleles with HBcAg-derived 9-mers at Pos 30 (residue number (starting from 0) of the peptide in the protein sequence), namely, the 9-mers core sequence LLDTASALY. Also, the prediction data of 9-mers sequence at Pos 29 (shifted forward by one peptide, DLLDTASAL) and Pos 31 (shifted backward by one peptide, LDTASALYR) were outputted as control. The score_EL of HLA-A*02:01, HLA-A*24:02, HLA-B*35:14, HLA-B*51:01, HLA-C*04:01, and HLA-C*16:02 with 9-mers at Pos 30 was 0.0264, 0.002041, 0.092112, 0.000118, 0.127962, and 0.013707, respectively. The % Rank_EL was 3.287, 8.205, 1.516, 50.286, 0.483, and 4.84, respectively. As a result, the bind level of HLA-B*35:14 and HLA-C*04:01 with 9-mers at Pos 30 was WB and SB, respectively. In contrast, only HLA-B*35:14 is a weak binder for 9-mers at Pos 29, which has the score_EL and % Rank_EL of 0.074915 and 1.812, respectively. Taken together, these results indicate that the 9-mers peptide LLDTASALY is enzymatically trimmed by ERAP-1 and followed by the presentation of predominantly HLA-C*04:01 molecule in HepG2.2.15 cells.

**TABLE 2:** Binding affinity prediction of 9-mers peptide with MHC-I molecules in HepG2.2.15 cells.

Pos	HLA alleles	Peptide	Core	Of	Gp	G1	IP	I1	Icore	Identity	% Rank_EL	Bind level
29	HLA-A*02:01	DLLDTASAL	DLLDTASAL	0	0	0	0	0	DLLDTASAL	Sequence	3.402	
30	HLA-A*02:01	LLDTASALY	LLDTASALY	0	0	0	0	0	LLDTASALY	Sequence	4.592	
31	HLA-A*02:01	LDTASALYR	LDTASALYR	0	0	0	0	0	LDTASALYR	Sequence	65	
29	HLA-A*24:02	DLLDTASAL	DLLDTASAL	0	0	0	0	0	DLLDTASAL	Sequence	13.027	
30	HLA-A*24:02	LLDTASALY	LLDTASALY	0	0	0	0	0	LLDTASALY	Sequence	8.205	
31	HLA-A*24:02	LDTASALYR	LDTASALYR	0	0	0	0	0	LDTASALYR	Sequence	60	
29	HLA-B*35:14	DLLDTASAL	DLLDTASAL	0	0	0	0	0	DLLDTASAL	Sequence	1.812	≤ WB
30	HLA-B*35:14	LLDTASALY	LLDTASALY	0	0	0	0	0	LLDTASALY	Sequence	1.516	≤ WB
31	HLA-B*35:14	LDTASALYR	LDTASALYR	0	0	0	0	0	LDTASALYR	Sequence	52.143	
29	HLA-B*51:01	DLLDTASAL	DLLDTASAL	0	0	0	0	0	DLLDTASAL	Sequence	3.802	
30	HLA-B*51:01	LLDTASALY	LLDTASALY	0	0	0	0	0	LLDTASALY	Sequence	13.068	
31	HLA-B*51:01	LDTASALYR	LDTASALYR	0	0	0	0	0	LDTASALYR	Sequence	50.286	
29	HLA-C*04:01	DLLDTASAL	DLLDTASAL	0	0	0	0	0	DLLDTASAL	Sequence	3.417	
**30**	HLA-C*04:01	**LLDTASALY**	**LLDTASALY**	**0**	**0**	**0**	**0**	**0**	**LLDTASALY**	**Sequence**	**0.48**	**≤ SB**
31	HLA-C*04:01	LDTASALYR	LDTASALYR	0	0	0	0	0	LDTASALYR	Sequence	42.8	
29	HLA-C*16:02	DLLDTASAL	DLLDTASAL	0	0	0	0	0	DLLDTASAL	Sequence	13.719	
30	HLA-C*16:02	LLDTASALY	LLDTASALY	0	0	0	0	0	LLDTASALY	Sequence	4.84	
31	HLA-C*16:02	LDTASALYR	LDTASALYR	0	0	0	0	0	LDTASALYR	Sequence	41.25	

Pos, Residue number (starting from 0) of the peptide in the protein sequence. MHC, specified MHC molecule/allele name. Peptide, amino acid sequence of the potential ligand. Core, the minimal 9 amino acid binding core directly in contact with the MHC. Of, the starting position of the Core within the Peptide (if > 0, the method predicts a N-terminal protrusion). Gp, position of the deletion, if any. Gl, length of the deletion, if any. Ip, position of the insertion, if any. Il, length of the insertion, if any. Icore, interaction core. This is the sequence of the binding core including eventual insertions of deletions. Identity, Protein identifier, i.e., the name of the FASTA entry. Score_EL, The raw prediction score. % Rank_EL, Rank of the predicted binding score compared to a set of random natural peptides. This measure is not affected by inherent bias of certain molecules toward higher or lower mean predicted affinities. Strong binders are defined as having % rank < 0.5, and weak binders with % rank < 2. We advise to select candidate binders based on % Rank rather than score bind level: (SB, strong binder; WB, weak binder). The peptide will be identified as a strong binder if the % Rank is below the specified threshold for the strong binders (by default, 0.5%). The peptide will be identified as a weak binder if the % Rank is above the threshold of the strong binders but below the specified threshold for the weak binders (by default, 2%). The bold value indicates that HLA-C*04:01 is a strong binder (SB) for the screened 9-mers peiptde.

## Discussion

The previous studies have shown that ERAP1 is dysregulated in autoimmune diseases (Burton et al., 2007), cancer (Wearsch and Cresswell, 2008; Cifaldi et al., 2011; James et al., 2013; Koumantou et al., 2019), and several types of viral disease including HIV (Tenzer et al., 2009), lymphocytic choriomeningitis virus (LCMV) (York et al., 2006), and human cytomegalovirus (HCMV) (Kim et al., 2011). In this study, both upregulation of ERAP1 and its secretion were observed in HBV-transfected HepG2.2.15 cells in contrast to germline HepG2 cells. Furthermore, the serum level of ERAP1 was significantly upregulated and moderately correlated with HBV DNA levels in patients with CHB. These results suggest that HBV infection results in ERAP1 expression both at cellular and organismic level. It is to be noted that both upregulation of ERAP1 and its secretion from the ER are induced by IFN-γ (Saric et al., 2002; Goto et al., 2011). However, it seems unlikely that ERAP1 upregulation in this study is elicited by IFN-γ as HBV infection has been reported to induce type-III but not type-I or type-II interferon. Furthermore, HBV has evolved various mechanisms to suppress IFN signaling (Mani and Andrisani, 2019). On the other hand, the previous study (Goto et al., 2011) has shown a supporting role of the secreted ERAP1 as it enhances the phagocytic activity of macrophages through the generation of active peptides. Thus, the intracellular and extracellular ERAP1 are functionally complementary and proportionally increased, as we have shown in Figures 1C,D. Collectively, these results suggested that HBV-mediated upregulation of ERAP-1 is involved in HBV-induced immune responses.

The proliferation of CD8^+^ T cells decreased significantly upon stimulated by HepG2.2.15 cells pretreated with ERAP1-IN-1 or ERAP1 gene knockdown (Figure 2). Furthermore, ERAP1-IN-1 treatment resulted in 6.24% and 4.32% reduction of the total numbers of peptides with 8 and 9 residues (Figure 3D), the length specific and predominant for ERAP1 trimming. These results indicated that ERAP1 promotes CD8^+^ T-specific antiviral immune response in the context of antigenic peptides presentation by MHC class I molecules. In contrast, the number of all peptides > 9-mers increased at the same time in comparison with the untreated group. These results were consistent with the previous observations in LCMV, HCMV, and vaccinia infection which showed an increase in the length of the viral peptides in the absence of ERAP1. Furthermore, a lack of antigen processing by ERAP1 profoundly altered the immune responses to LCMV, HCMV, and vaccinia virus, characterized by the decreased frequency of CD8^+^ T cells specific for viral epitopes (York et al., 2006; Blanchard et al., 2010). Overall, these results confirm the unique enzymatic properties of ERAP1 in HBV-infected HepG2.2.15 cells, leading to precise trimming of longer peptides to 8–9-mers length. Furthermore, the final peptides trimmed by ERAP1 were presented by MHC class I molecule to induce CD8^+^ T-cell-mediated antiviral immune responses.

As the present studies demonstrate, three kinds of 8–16 residues peptides derived from two HBV antigens, HBsAg and HBcAg, were observed in HepG2.2.15 cells. The 8-mers (161–168, FLWEWASV) peptide is cleaved from HBsAg, and the 9-mers (30–38, LLDTASALY) and 15-mers (41–55, ALESPEHCSPHHTAL) peptides were enzymatically cut from HBcAg. The inhibitor ERAP1-IN-1 produced a complete block of 9-mers and a substantial increment of 15-mers while having no remarkable effect on 8-mers, suggesting that ERAP1 trims HBcAg-specific peptides in HBV-infected cells. The HBV C gene contains two in-phase initiation codons, the C region and the pre-C sequence, which directs the synthesis of HBcAg (185 aa) and of a pre-core protein which upon processing results in the secretion of HBeAg (159 aa) (Jean-Jean et al., 1989, p. 30). Multiple copies of HBcAg dimers, mainly the N-terminal 155 aa, form HBV nucleocapsids which endow the viral particles with high immunogenicity (Gallina et al., 1989). The previous study has shown that 4 mutants at position A11-E8, L37-A41, R39-L31, and A41-E43 of HBcAg were not able to assemble detectable amounts of capsids in co-transfected HuH7 cells (Koschel et al., 2000). Thus, it is very likely that capsid formation is a prerequisite for trimming of HBcAg by ERAP1 since the 9-mers (30-38, LLDTASALY) peptide is located within the above-mentioned mutation region. Immunization studies have demonstrated that HBsAg and HBcAg are capable of eliciting distinct, humoral, and cell-mediated immune responses, respectively (Hoofnagle et al., 1975). Apparently, the HBcAg-derived 9-mers peptide is loaded in the ER by MHC class I molecules, which are eventually presented as peptide-MHC I (pMHC I) complexes on the cell surface to induce CD8^+^ T-cell-mediated antiviral immune responses. On the other hand, we also observed a substantial increment of 15-mers peptide upon ERAP1-IN-1 treatment. However, the 8–9-mers enzymatic fragments of the increased peptide were not observed in untreated control. It will be useful to determine whether hyper-editing of the 15-mers peptide happened as a proportion of peptides that enter the ER are destroyed by ERAP1 to below the minimal size needed for presentation on MHC class I molecules (Hammer et al., 2007; Georgiadou and Stratikos, 2009; Chen and Tian, 2019). Therefore, ERAP1 functions in a paradoxical manner as an interrupter limiting antigen presentation while it is still an ideal aminopeptidase to generate the final optimal length of peptides for pMHC I presentation (York et al., 2002).

The predictive analysis by the NetMHCpan-4.1 server showed that HLA-C*04:01 molecule is a strong binder for the 9-mers peptide LLDTASALY, which is enzymatically trimmed by ERAP-1 in HepG2 cells. In addition, HLA-B*35:14 was observed to be a weak binder for both 9-mers at Pos 30 and Pos 29. The previous study has shown that HLA-C*04:01 is one of the most frequent HLA class I alleles in Cameroonian infected with HBV, with a frequency of 37.5% in patients with HBV compared with that of 25.5% in healthy populations (Yengo et al., 2020). In addition, HLA-C*04:01 was also observed to be in association with poor viral control of HIV (Olvera et al., 2015) and severe clinical course of COVID-19 (Weiner et al., 2021). However, the genome-wide association study (GWAS) findings highlighted the importance of HLA-C in the clearance of HBV infection in addition to HLA-DP and HLA-DQ (Hu et al., 2013). Thus, accurate functional and genetic study is needed to ascertain the correlation between HLA-C*04:01 and HBV infection.

## Conclusion

In conclusion, our findings identify a previously unknown viral antigen peptides presentation-based immune response mechanism that targets a key step in the MHC class I antigen-processing pathway.

## Data Availability Statement

The data presented in the study are deposited in the ProteomeXchange Consortium repository (http://proteomecentral.proteomexchange.org), accession number PXD031858.

## Ethics Statement

The studies involving human participants were reviewed and approved by Ethics Committee for the Second Affiliated Hospital of Anhui Medical University. The patients/participants provided their written informed consent to participate in this study.

## Author Contributions

HL and LC: conceptualization. HL and BH: data curation. JH, QW, and FW: formal analysis. HL, BH, and JH: investigation. HL: writing—original draft. LC and FP: writing, reviewing, and editing. All authors have read and agreed to the published version of the manuscript.

## Conflict of Interest

The authors declare that the research was conducted in the absence of any commercial or financial relationships that could be construed as a potential conflict of interest.

## Publisher’s Note

All claims expressed in this article are solely those of the authors and do not necessarily represent those of their affiliated organizations, or those of the publisher, the editors and the reviewers. Any product that may be evaluated in this article, or claim that may be made by its manufacturer, is not guaranteed or endorsed by the publisher.

## Footnotes

1
http://proteomecentral.proteomexchange.org

2
http://www.cbs.dtu.dk/services/NetMHCpan/

3
https://web.expasy.org/cellosaurus/CVCL_0027

## Supplementary Material

Supplementary Figure 1 ERAP1-IN-1 displays no toxicity to HepG2.2.15 cells at working concentration (50 μm). Cell viability of HepG2.2.15 cells treated with gradient concentrations of ERAP1-IN-1 was assayed by WST-8/CCK-8, and the positive control group (cells left untreated) was normalized to 100%.

Supplementary Figure 2 Titration of ERAP1-IN-1 inhibits recombinant ERAP1 activity *in vitro*.

Supplementary Figure 3 The level of HBsAg, HBeAg, and HBV-DNA in the supernatants of HepG2.2.15 cells treated or left untreated with ERAP1-IN-1 for 72 h.
