# Erratum: Endoplasmic reticulum aminopeptidase 1 is involved in anti-viral immune response of hepatitis B virus by trimming hepatitis B core antigen to generate 9-mers peptides

**DOI:** 10.3389/fmicb.2023.1273875

**Published:** 2023-08-18

**Authors:** Frontiers Production Office

**Affiliations:** Frontiers Media SA, Lausanne, Switzerland

**Keywords:** hepatitis B, endoplasmic reticulum aminopeptidase 1, major histocompatibility complex class I, antigen presentation, immune response

Due to a production error, Reviewer 1 was erroneously named in the published article. The name of Reviewer 1 has been removed.

The publisher apologizes for this mistake. The original article has been updated.

