# Supplementary figures and images for: Endoplasmic Reticulum Aminopeptidase 1 Is Involved in Anti-viral Immune Response of Hepatitis B Virus by Trimming Hepatitis B Core Antigen to Generate 9-Mers Peptides
Source: Front Microbiol. 2022 May 4;13:829241. doi: 10.3389/fmicb.2022.829241 (PMC9115554; doi:10.3389/fmicb.2022.829241)

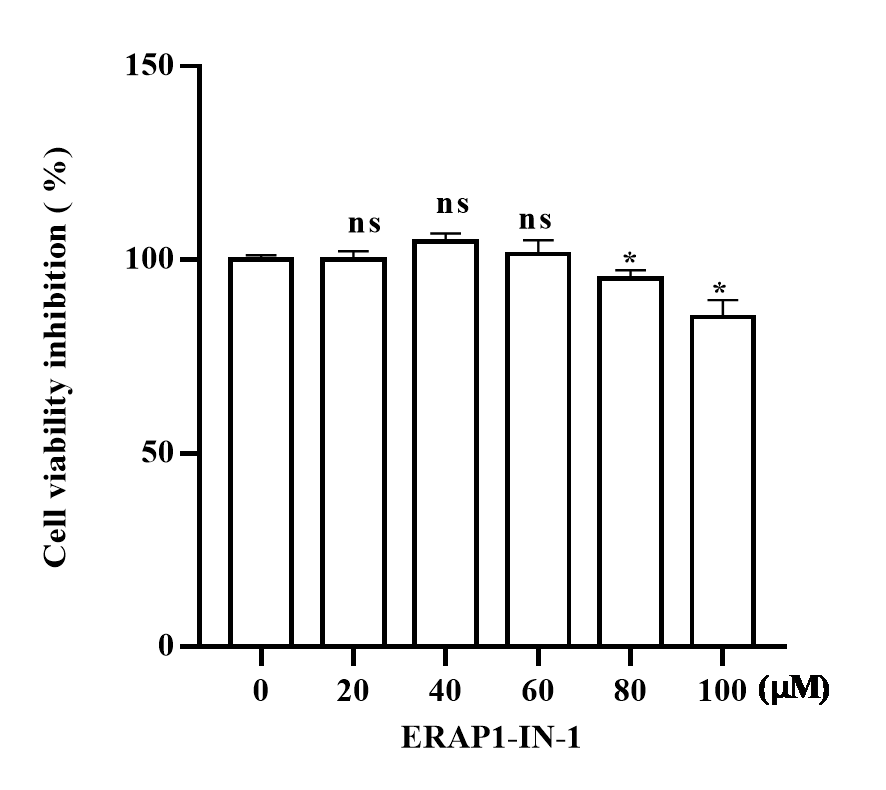

Supplement: Supplementary Figure 1 — ERAP1-IN-1 displays no toxicity to HepG2.2.15 cells at working concentration (50 μm). Cell viability of HepG2.2.15 cells treated with gradient concentrations of ERAP1-IN-1 was assayed by WST-8/CCK-8, and the positive control group (cells left untreated) was normalized to 100%. [file Image_1.TIF]

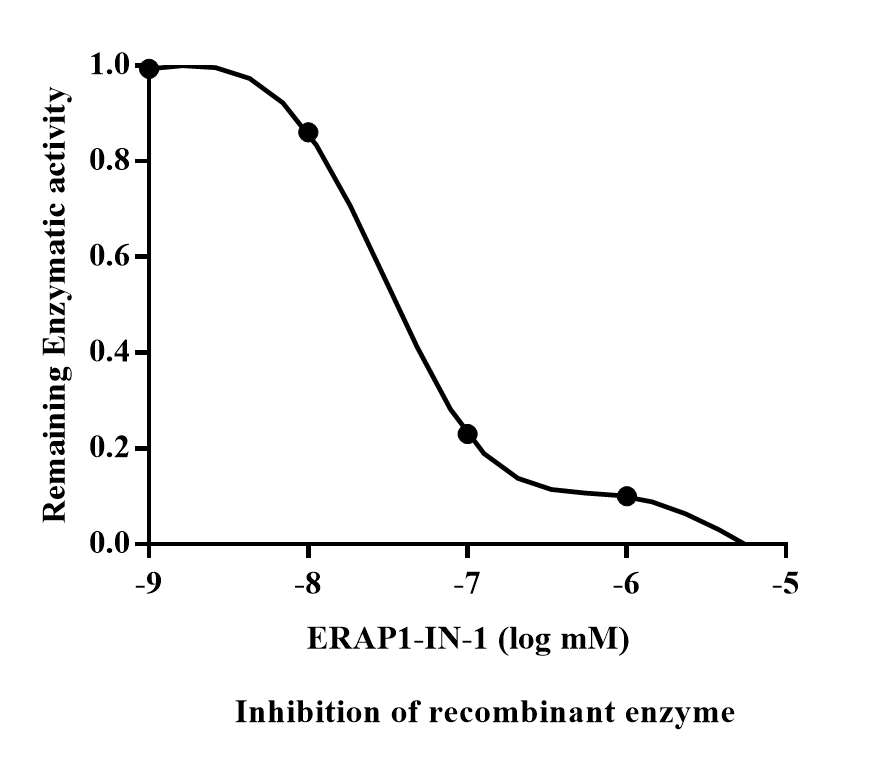

Supplement: Supplementary Figure 2 — Titration of ERAP1-IN-1 inhibits recombinant ERAP1 activity in vitro. [file Image_2.TIF]

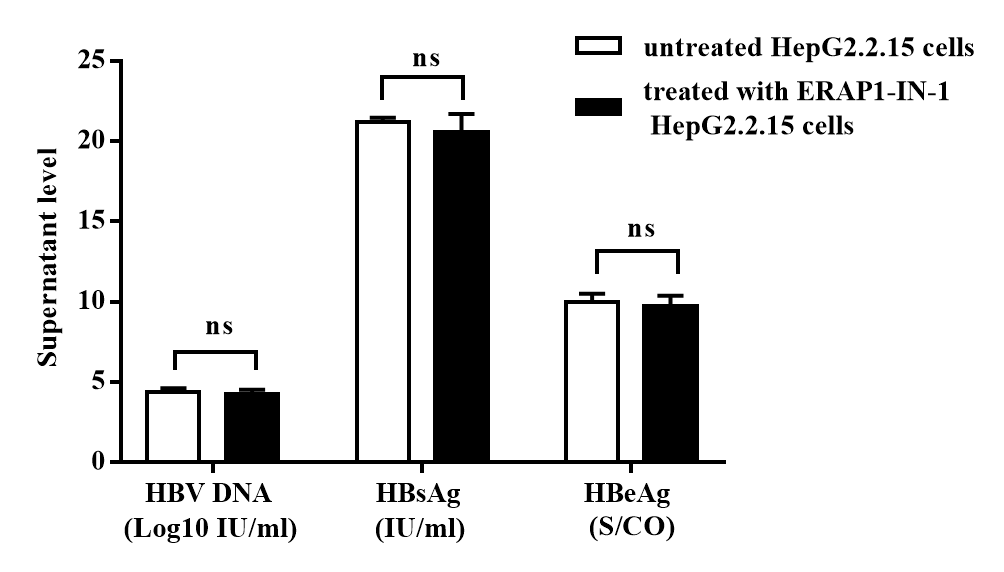

Supplement: Supplementary Figure 3 — The level of HBsAg, HBeAg, and HBV-DNA in the supernatants of HepG2.2.15 cells treated or left untreated with ERAP1-IN-1 for 72 h. [file Image_3.TIF]
